# Exome sequencing of deer mice on two California Channel Islands identifies potential adaptation to strongly contrasting ecological conditions

**DOI:** 10.1002/ece3.8357

**Published:** 2021-11-17

**Authors:** John L. Orrock, Linelle Abueg, Stephen Gammie, Jason Munshi‐South

**Affiliations:** ^1^ Department of Integrative Biology University of Wisconsin Madison Wisconsin USA; ^2^ Louis Calder Center – Biological Field Station Fordham University Armonk New York USA

**Keywords:** antipredator behavior, exome, genotype‐by‐environment association, immunity, islands, signatures of selection

## Abstract

Understanding the forces that drive genotypic and phenotypic change in wild populations is a central goal of evolutionary biology. We examined exome variation in populations of deer mice from two of the California Channel Islands: *Peromyscus maniculatus elusus* from Santa Barbara Island and *P. m. santacruzae* from Santa Cruz Island exhibit significant differences in olfactory predator recognition, activity timing, aggressive behavior, morphology, prevalence of Sin Nombre virus, and population densities. We characterized variation in protein‐coding regions using exome capture and sequencing of 25 mice from Santa Barbara Island and 22 mice from Santa Cruz Island. We identified and examined 386,256 SNPs using three complementary methods (BayeScan, pcadapt, and LFMM). We found strong differences in molecular variation between the two populations and 710 outlier SNPs in protein‐coding genes that were detected by all three methods. We identified 35 candidate genes from this outlier set that were related to differences in phenotypes between island populations. Enrichment analyses demonstrated that patterns of molecular variation were associated with biological processes related to response to chemical stimuli and regulation of immune processes. Candidate genes associated with olfaction (*Gfy*, *Tlr2*, *Vmn13r2*, numerous olfactory receptor genes), circadian activity (*Cry1*), anxiety (*Brca1*), immunity (*Cd28*, *Eif2ak4*, I*l12a*, *Syne1*), aggression (*Cyp19a*, *Lama2*), and body size (*Bc16*, *Syne1*) exhibited non‐synonymous mutations predicted to have moderate to large effects. Variation in olfaction‐related genes, including a stop codon in the Santa Barbara Island population, suggests loss of predator‐recognition traits at the molecular level, consistent with a lack of behavioral aversion to fox feces. These findings also suggest that divergent pathogen prevalence and population density may have influenced adaptive immunity and behavioral phenotypes, such as reduced aggression. Overall, our study indicates that ecological differences between islands are associated with signatures of selection in protein‐coding genes underlying phenotypes that promote success in those environments.

## Graphical abstract

Understanding the forces that drive genotypic and phenotypic change in wild populations is a central goal of evolutionary biology. We examined molecular variation in mice from two different islands that differ greatly in predators, pathogens, and competitors and found significant molecular variation in genes for olfactory predator recognition, activity timing, immunity, and aggression. These findings demonstrate the molecular foundation of selection‐mediated differences among organisms, and also help identify important genes for future studies to investigate adaptive evolution.

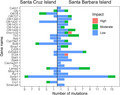

## 1 INTRODUCTION

A primary goal of evolutionary biology is to understand how selection gives rise to genetic and phenotypic variation. Islands provide an outstanding opportunity to understand how ecological factors such as resource availability (Grant & Grant, 1993), competitive interactions (Grant & Grant, 1993), and predators (Blumstein & Daniel, 2005; Losos et al., 2006) affect evolution. Island systems also provide an opportunity to understand the ecological and evolutionary implications of novel conditions, as island taxa often experience dramatic declines when novel competitors, predators, or diseases are introduced (Russell & Kueffer, 2019). Given that natural systems across the world are experiencing novel climatic variation (Turner et al., 2020), novel predators (Guiden et al., 2019), and novel pathogens (e.g., COVID‐19), island systems may also be particularly promising for understanding the targets of selection and subsequent evolutionary responses to new evolutionary pressures in the Anthropocene.

The insights gleaned from island systems gain additional utility when species found in island settings are also widely distributed in mainland settings, because phenotypic and molecular evolution revealed on islands provides context for understanding changes in different environmental contexts. For example, deer mice (*Peromyscus maniculatus*) are found across much of North America, including several island archipelagos (Bedford & Hoekstra, 2015). A high‐quality reference genome also exists for *P. maniculatus* (Bendesky et al., 2017) and other *Peromyscus* species (Long et al., 2019), making the study of molecular and phenotypic variation in *P. maniculatus* particularly promising for gaining insights into which loci are likely to be under strong selection, in which portions of the genome are likely to evolve in particular ecological settings, and for identifying the genotypic basis of key phenotypic traits (Linnen et al., 2013; Storz et al., 2012).

In this paper, we evaluate whether differences in exposure to predators, pathogens, and competitors are associated with underlying exome variation between *P. maniculatus* on two of the California Channel Islands; although recent revisions imply that Channel Islands mice may be subspecies of *P. gambelii*, this work did not directly examine Channel Islands specimens (Bradley et al., 2020). As such, we use *P. maniculatus* here to remain consistent with past literature until additional taxonomic research is conducted. The Channel Islands provide an ideal opportunity to examine mammalian evolution in several taxa, including endemic island foxes (Funk et al., 2016) and spotted skunks (Floyd et al., 2011). Deer mice on the Channel Islands have been isolated from mainland populations for 8000–12,000 years (Ashley & Wills, 1987; Durst, 2014), have undergone rapid morphological evolution (Pergams & Ashley, 1999), and exhibit considerable morphological and genetic variation (Ashley & Wills, 1987; Durst, 2014; Gill, 1980).

The considerable phenotypic variation observed among mice from different islands may reflect differences in selection pressures among islands (Table 1). For example, *P. maniculatus elusus* on Santa Barbara Island have not experienced fox predators for 10,000–12,000 years (Durst, 2014) and do not exhibit aversion to olfactory fox cues (Orrock, 2010) In contrast, *P. maniculatus santacruzae* from San Miguel island have experienced fox predation for at least 7100 years (Funk et al., 2016) and exhibit avoidance of olfactory fox cues (Orrock, 2010). Mice on Santa Barbara Island are also active earlier in the evening (Table 1), which may reflect the lack of crepuscular predators (foxes) on that island (Connolly & Orrock, 2018). Islands also differ in the prevalence of zoonotic pathogens: The prevalence of Sin Nombre virus in mice on Santa Cruz Island can be as high as 71% (Graham & Chomel, 1997), while Sin Nombre virus has never been documented on Santa Barbara Island despite more than two decades of sampling (Graham & Chomel, 1997; Orrock et al., 2021). Population dynamics are also different between the islands, with populations on relatively predator‐free Santa Barbara Island achieving very high densities and cyclic dynamics compared to populations on larger islands with more diverse predator communities, which have non‐cyclic (although still seasonal) population densities similar to mainland populations (Drost & Fellers, 1991; Drost et al., 2009). Despite higher densities, mice from Santa Barbara Island exhibit less wounding (Table 1), suggesting differences in aggressive behavior potentially as a result of high population densities (Baier & Hoekstra, 2019; Halpin & Sullivan, 1978). Body size and morphological characteristics also differ among islands (Gill, 1980; Pergams & Ashley, 1999), including significantly longer tails and smaller ears on Santa Cruz Island than on Santa Barbara Island (Gill, 1980). While environmental and phenotypic differences suggest the potential for molecular evolution of mouse populations on the California Channel Islands, our knowledge of molecular differences between island populations is limited to studies of allozymes, RFLPs, and microsatellites (Ashley & Wills, 1987; Durst, 2014; Gill, 1980). While these previous studies have been valuable for documenting genetic variation, our ability to fully appreciate the scope of these molecular changes is hampered by their necessary focus on a few loci and the inability to identify important genetic variants experiencing selection pressures.

**TABLE 1:** Summary of the phenotypic traits where differences in phenotype have been documented between wild mice on two of the California Channel Islands: *Peromyscus maniculatus elusus* on Santa Barbara Island (SBI) and *P. maniculatus santacruzae* found on Santa Cruz Island (SCI)

Phenotype	Difference in phenotype between islands and putative selective cause
Olfactory predator recognition	Mice on SBI do not avoid olfactory cues of foxes, but *P. m. santacruzae* from a fox‐containing island once connected with SCI (San Miguel island) exhibit avoidance of olfactory fox cue (Orrock, 2010). Lack of avoidance on SBI may be because foxes not present on SBI but present on SCI (Orrock et al., 2011).
Activity timing	Lack of foxes on SBI and high population densities may select for earlier foraging; individuals on SBI are active earlier in the day than those on SCI (Orrock, unpublished data)
Immunity	Sin Nombre virus has never been found in SBI population; SNV prevalence in SCI population can be very high (Graham & Chomel, 1997; Orrock et al., 2021).
Increased stress and anxiety	Higher population densities observed on SBI (Drost & Fellers, 1991; Drost et al., 2009) and greater aggression observed among SCI mice (higher rates of ear injury; (J. L. Orrock, unpublished data) compared with SBI mice.
Growth and body size	SBI mice have lower mass and shorter tails than mice from SCI (Gill, 1980; Pergams & Ashley, 1999), possibly due to differences in predation pressure, abiotic resources, or intraspecific competition

Note

Data sources are indicated in parentheses. When no source is indicated, the data come from *P. maniculatus* individuals used in this study.

In this paper, we use exome capture to evaluate differences in protein‐coding genes of *Peromyscus maniculatus* from populations on two islands that exhibit marked differences in olfaction, immunity, activity timing, aggression, and body size (Table 1). We sampled *P. maniculatus* from two of the California Channel Islands, *P. m. santacruzae* from Santa Cruz Island and *P. m. elusus* from Santa Barbara Island (Table 1). Neither of these islands have ever been connected to the mainland (Durst, 2014). Molecular evidence suggests that *Peromyscus maniculatus* have been on Santa Cruz for at least 12,000 years (Durst, 2014), when Santa Cruz Island, Santa Rosa Island, San Miguel Island, and Anacapa Island were all a single island (Santarosae) due to lower sea level. Molecular studies suggest that *P. m. elusus* on Santa Barbara Island are likely descended from mice from Santarosae (Ashley & Wills, 1987; Durst, 2014). Our objective was to identify signatures of selection associated with molecular divergence between the two island populations and to evaluate whether molecular variation is consistent with observed variation in mouse phenotypes (Table 1). We begin by identifying differences in allele frequencies between the two island populations using Fst‐based and principal component‐based selection scans (Foll & Gaggiotti, 2008; Luu et al., 2017), as well as genotype‐by‐environment association tests (Frichot & François, 2015). Then, we evaluate gene functions and gene ontology enrichment results to determine whether variants under selection are potentially associated with phenotypic differences between the islands.

## 2 METHODS

### 2.1 Field methods

We collected tissue samples from 25 live‐caught deer mice on Santa Barbara Island and 22 mice from Santa Cruz Island from July 26 to July 31, 2016. Mice were captured in Sherman Live traps baited with rolled oats. Upon capture, age, sex, reproductive status, and mass were recorded. We removed ~1mm of the tail tip for a tissue sample (Orrock, 2021). Mice were released at the site of capture after sampling. Samples were stored in a −20C freezer until processing in the laboratory; all field procedures were approved by the University of Wisconsin RARC (Protocol L005041).

### 2.2 Exome capture for high‐throughput DNA sequencing

Arbor Biosciences generated the exome capture bait set using the coding sequence reference from the *P. maniculatus* v. 1.0 genome (NCBI GCA_000500345.1: https://www.ncbi.nlm.nih.gov/assembly/GCA_000500345.1). After best‐hit genomic intervals were padded to a minimum of 80 nucleotides and collapsed to contiguous intervals, the space totaled approximately 43.5 Mbp of target space. These regions were used to design probes (80 nucleotide length) spaced about every 100 base pairs across the target space. To eliminate baits that might hybridize, Arbor Biosciences then used their “ultra‐stringent” filtration method to filter probe candidates for single‐locus specificity, with additional filtration of any candidate whose best hit was 13% or more soft‐masked. This resulted in a final bait set of 378,989 unique bait sequences, which were then synthesized as part of a custom myBaits^®^ kit.

Genomic DNA from tail snips was extracted using Qiagen DNeasy Blood & Tissue Kits following the standard protocol. DNA extracts were provided to Arbor Biosciences for library preparation, target enrichment, and sequencing as part of a myReads^®^ next‐generation sequencing project. After quality assessment, genomic DNA was sonicated using a QSonica Q800R instrument and size‐selected using SPRI beads to average lengths of 300 base pairs. Each sonicated, size‐selected sample was then converted to an Illumina^®^ TruSeq‐style sequencing library, which included amplification for 7 cycles to add dual indexes in unique combinations. Up to 200 nanograms of each sample library was combined into pooled libraries of up to 8 sample libraries. These pools were enriched using the custom myBaits^®^ kit following the myBaits^®^ manual version 3.2 protocol. After hybrid capture and cleanup, bead‐bound enriched library pools were amplified using universal primers for an additional 10 cycles before purification and sequencing. The 19 enriched pools were then sequenced across 6 lanes of the Illumina^®^ HiSeq^®^ 2500 platform using PE125 sequencing protocol, and reads were de‐multiplexed using CASAVA^®^ and the unique index pairs, while tolerating a 1‐nucleotide mismatch.

### 2.3 Reference genome alignment and SNP calling

Adaptor and end trimming were performed with trim_galore before sequencing reads for each individual were aligned to the *Peromyscus maniculatus* v. 1.0 genome using default parameters in bwa mem (Martin, 2011). The average per‐individual coverage over our targeted regions was 22.9X (SD = 9.3), the median was 22.1X, and the range was 4.1X–54.2X. Single nucleotide polymorphisms (SNPs) were called using the Genome Analysis Toolkit (GATK version 3.9) following the pipeline in Pfeifer et al. (2018) and general filtering guidelines from the Broad Institute (DePristo et al., 2011; McKenna et al., 2010; Pfeifer et al., 2018; Van der Auwera et al., 2013). SAMtools, GATK, and Picard were used to generate the final collective VCF using GATK’s gVCF pipeline.

HaplotypeCaller was used to create individual gVCF files, a GATK file format that denotes blocks of reference sequence and SNPs for an individual. The resulting 47 gVCF files were then jointly genotyped using the GenotypeGVCFs walker, resulting in one VCF with biallelic and multiallelic SNPs, as well as insertions and deletions (indels). Indels and multiallelic SNPs were removed before proceeding with SNP filtration. SNPs were filtered following Broad Institute's general hard filtering recommendations: MappingQuality < 40.0, QualByDepth < 2.0, MappingQualityRankSumTest < −12.5, and ReadPosRankSumTest < −8.0. Remaining SNPs were filtered to retain SNPs with minor allele frequencies >5% and no missing data.

While most alignments with the *Peromyscus* genome identified genes with an official gene symbol, a subset was best matched with a locus (e.g., LOC102919364). For significant LOC genes, we used the Gene tool within NCBI and ran BLAST against the house mouse (*Mus musculus*) genome to gain additional information. None of the olfactory receptors that differed between islands had an official gene symbol, so these were further evaluated using the BLAST function and identified with the closest house mouse olfactory receptor (based on coverage and percent identical).

### 2.4 Outlier detection

We used three different analytical approaches to identify outlier SNPs between the two island populations. BayeScan (version 2.1) uses a reversible‐jump Markov chain Monte Carlo approach to identify F_ST_ outliers given priors such as population assignment. BayeScan 2.1 was run using default parameters aside from a prior odds value of 2, corresponding to neutrality being twice as likely as selection. Default parameters include thinning interval = 10, 20 pilot runs of 5000, and a burn‐in length of 50,000. We ran BayeScan with two prior populations defined, corresponding to the two islands. For outlier detection, a false discovery rate of 0.05 was used.

*Pcadapt* detects outlier loci based on principal component analysis and does not rely on prior population assignments. Outliers identified by *pcadapt* contribute to population structure much more strongly than average SNPs. We report results from *pcadapt* using a K = 2, which was chosen after running *pcadapt* for K = 1–20 and evaluating the screeplot. Q‐values were determined using the *qvalue* R package as described in the *pcadapt* vignette, and a q‐value cutoff of 0.0001 was used. We used LFMM (latent factor mixed modeling) to conduct genotype‐by‐environment association tests where source island was a binary environmental variable using the R package LEA. LFMM detects outliers by identifying significant associations between SNP allele frequencies and environmental gradients or phenotypic traits, after accounting for population structure. LEA’s ancestry analysis, sNMF, indicated a most likely K value of 2, which was used for subsequent LFMM analysis. We ran LFMM for 10 repetitions, and p‐values were adjusted using a value of 1. A q‐value cutoff of 0.05 was used for outlier detection. We used pcadapt and LFMM because they account for population structure when identifying outliers. Population structure between the two islands could make it difficult to distinguish between signatures caused by drive vs. selection, but using multiple approaches that account for structure should minimize false‐positive outliers.

After annotating our SNP list to obtain a list of genes containing outlier SNPs, we selected a subset of individual genes to evaluate shifts in allele frequency and change in gene function between the two island populations. These genes were selected based upon their association with phenotypic traits that appear to differ between the islands, that is, genes with published evidence that they are associated with olfaction, immune function, growth and size, activity timing, or stress, anxiety, and aggression (Table 2). Given documented rapid morphological evolution in mice from these islands (Pergams & Ashley, 1999), we also evaluated genes associated with growth and body size. Genes were identified by checking outlier SNP coordinates against the *P. maniculatus* Generic Feature Format 3 (GFF3) file. Outlier SNPs that fell within genic regions were separately retained for each analysis. Per‐population allele frequencies for retained outlier SNPs were calculated in VCFtools. VariantEffectPredictor was used to determine which SNPs might have a greater effect on coding, based off the potential amino acid change or introduction of stop or splice elements (Ensembl) (Yates et al., 2020).

**TABLE 2:** Summary of candidate genes and functions

Symbol	MGI Gene ID	Phenotypic relevance				
		Olfaction	Immune function	Growth and size	Activity timing	Stress, anxiety, aggression
*Calm1*	MGI:88251	X			X	
*Gfy*	MGI:2685427	X				
Olfr76*	MGI:2153205	X				
Olfr177*	MGI:3030011	X				
Olfr196*	MGI:3030030	X				
Olfr206*	MGI:3030040	X				
Olfr215*	MGI:3030049	X				
Olfr727*	MGI:3030561	X				
Olfr1340*	MGI:3031174	X				
Olfr1420*	MGI:3031254	X				
Olfr1431*	MGI:3031265	X				
Olfr1434*	MGI:3031268	X				
Olfr1440*	MGI:3031274	X				
Vmn1r32*	MGI:2159451	X				
Tlr2*	MGI:1346060	X	X	X	X	
*App*	MGI:88059		X	X	X	X
Cyp19a1*	MGI:88587		X	X		X
*Nf1*	MGI:97306		X	X		
*Bcl6*	MGI:107187		X			
*Brca1*	MGI:104537		X			X
*Cd28*	MGI:88327		X			
*Eif2ak4*	MGI:1353427		X			
*Il12a*	MGI:96539		X			
*Il15*	MGI:103014		X	X		
*Stat1*	MGI:103063		X			
*Stat4*	MGI:103062		X			
*Cacna2d1*	MGI:88295			X		X
*Dcc*	MGI:94869			X		
*Lama2*	MGI:99912			X		X
*Mmp24*	MGI:1341867			X		
*Ryr1*	MGI:99659			X	X	
*Syne1*	MGI:1927152			X	X	X
*Wfs1*	MGI:1328355			X		X
*Cry1*	MGI:1270841				X	
*Smarca4*	MGI:88192					X

Note

Gene symbols for olfactory receptors in mice were taken from the mouse gene closest to the *Peromyscus* BLAST result (indicated by *).

### 2.5 Functional analysis of genes associated with outlier SNPs

For functional analysis, we entered all identified, annotated outlier genes into STRING (Szklarczyk et al., 2019). STRING is a protein–protein interaction tool, which identifies empirically validated or predicted interactions between proteins based on a wide array of databases, and with this tool, we identified top interacting proteins (from among the annotated genes) with an interaction score of 0.40 or greater between any two genes as a cutoff. We then ran this approach a second time and identified 77 genes that interacted with at least ten other genes in the reduced list. These genes were replotted in STRING and analyzed using the enrichment tool. The rationale for this analysis is that highly interactive genes could synergize to have an oversized effect on phenotype.

## 3 RESULTS

Our dataset contained 386,356 SNPs; evolutionary clustering analysis conducted with all SNPs in Admixture, as well as F_ST_ values, indicated strong divergence between the two island populations (Figures S1 and S2). Of these SNPs, BayeScan identified 10,913 outlier SNPs (5.1% of total) within 1430 unique genes and *Pcadapt* identified 16,212 outlier SNPs (4.2%) within 2592 unique genes. For LFMM, we retained the top 1% of outliers, which amounted to 2957 SNPs within 1097 unique genes. 803 SNPs (0.2%) were identified as outliers by all three methods. Of these, 709 were associated with annotated genes (Table S1).

**FIGURE 1:**
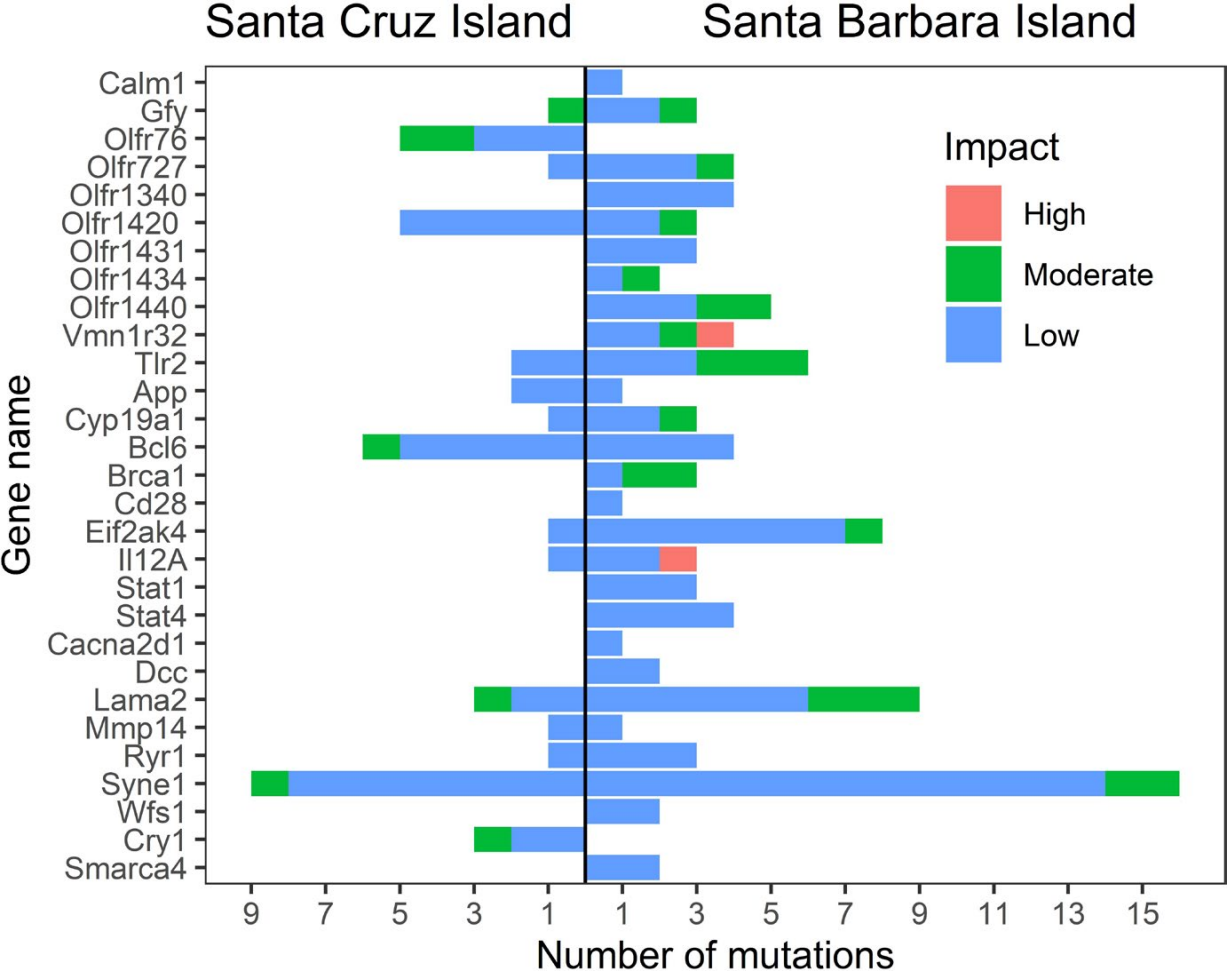
Overview of SNP variation within candidate genes and the predicted phenotypic impact of each polymorphism. Only mutations with an Ensembl impact of low, moderate, or high are listed; mutations with a modifier impact (e.g., mutations that produce an intergenic variant, an upstream gene variant) are not listed. As a result, genes where mutations were only modifiers (*Il15*, *Nf1*, *Olfr177*, *Olfr196*, *Olfr206*, *Olfr215*) are not listed in the figure

Using the outlier SNPs identified by all three methods, we evaluated annotated genes associated with these outlier SNPs and chose 35 genes to evaluate further based on their association with phenotypes of a priori interest: olfactory ability, immune function, growth and size, activity timing, or stress, anxiety, and aggression (Table 2). A total of 563 variants were present in the SNPs associated with the 35 candidate genes. Of these, 420 (74.6%) were either intron or intergenic variants, while 143 variants were labeled by variant effect predictor as variants of low, moderate, or high impact, depending upon the degree to which they are expected to affect gene function (e.g., synonymous variants are considered low‐impact variants, while stop codons are considered high‐impact variants). 80 synonymous mutations (low‐impact variants) were in the Santa Barbara Island population and 35 in the Santa Cruz Island population (Figure 1). All of the non‐synonymous mutations (moderate‐impact variants impact) found were missense variants, where the mutation leads to a change in the amino acid sequence, but the length of the transcript is preserved. These were more common in the Santa Barbara Island population (19 variants) compared with the Santa Cruz Island population (7 variants). We found two mutations predicted to have a high impact (gained stop codon), and both were in the Santa Barbara Island population (*Vmn1r32* and *Il12a*; Figure 1). In accordance with the strong divergence indicated by analysis of all SNP data (Figures S1 and S2), the majority of alleles (516 of 563; 91.7%) in SNPs associated with the 35 candidate genes were fixed between island populations. For the 49 mutations that were not fixed, allele frequencies were all within 5% of fixation (Table S2).

**FIGURE 2:**
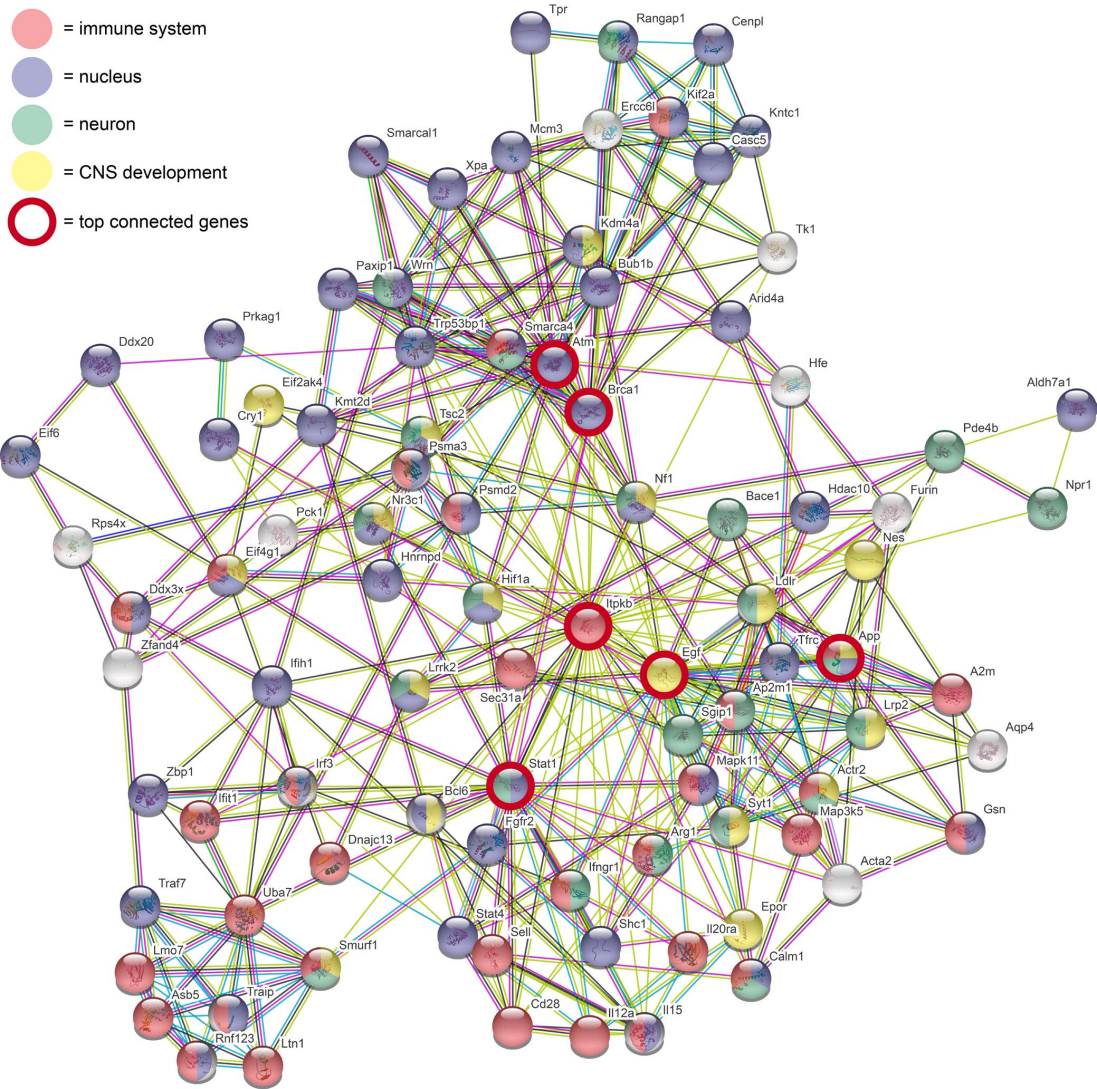
Top interconnected genes. Beginning with all 709 outlier genes, STRING was used to identify the top interaction genes based on a variety of published evidence. Circles are genes, and a line (edge) between genes indicates a known connection. Circles are colored according to known function, including immune system (red), nucleus (blue), neuron (green), and CNS development (yellow). Color of line indicates type of evidence as follows: light blue (known interactions from curated databases); pink (known interactions that are experimentally determined); green (predicted interactions from gene neighborhood); red (predicted interactions from gene fusions); blue (predicted interactions from gene co‐occurrence); light green (text mining); black (co‐expression); and gray (protein homology). The very top interacting genes are highlighted with a bold red circle

Enrichment analysis of GO terms associated with non‐synonymous mutations in the 709 annotated gene outlier SNPs identified overrepresentation of the immune system, nervous system development, the nucleus, and the neuron (Tables S3–S5). Twelve significant GO terms in the Biological Process category were related to the immune system and were associated with 105 genes in our analysis. One gene, *Itpkb*, was not recognized by the enrichment tool but was added to the list due to its known involvement in the immune response. Thus, 106 of the 709 evaluated genes relate to immune function. Using STRING, we identified 77 of the 709 genes that strongly interacted with one another (Figure 2). The gene with the highest level of interaction was *Itpkb*, followed by *Egf*, *Stat1*, *App*, *Brca1*, and *Atm*. Many of these genes are involved in the immune system, gene expression, nervous system development, and the MAP kinase pathway (Figure 2). A number of the genes, including *App* and *Hif1a*, are involved in multiple processes.

## 4 DISCUSSION

We find molecular variation indicating substantial adaptive divergence between *P. maniculatus* subspecies on two ecologically contrasting California Channel Islands. Particular genetic variants we identified are related to phenotypic variation previously measured between rodent populations on the two islands. Our findings suggest that differences in predator guilds, pathogens, and population density may have contributed to significant differences in phenotypic (Table 1) and genotypic (Figures 1 and 2, Table 2) variation between these two island populations.

Significant differences in olfactory genes may provide additional evidence supporting the loss of predator‐recognition behavior by mice on Santa Barbara Island. Previous behavioral studies have found that mice on Santa Barbara Island (which has lacked foxes or skunks for 12,000 years) did not respond to olfactory cues of foxes (feces), while *P. m. santarosae* from San Miguel Island did exhibit aversion to fox cues (Orrock, 2010). Our results suggest that this phenotypic variation may be reflected in differences in SNPs related to olfactory genes. The mouse population on Santa Barbara Island was characterized by a greater number of non‐synonymous mutations for olfaction‐related genes, including a loss‐of‐function mutation for vomeronasal type 1 receptor (*Vmn1r32*), as well as non‐synonymous mutations in genes with a known role in olfaction (e.g., Toll‐like receptor 2, *Tlr2*). One gene with a key role in olfaction, *Gfy* (Golgi‐associated olfactory signaling regulator), exhibits mutations of moderate effect on both islands. Such patterns of non‐synonymous mutations that differ among islands may signal the role of different processes on each island, such as relaxed selection on one island (i.e., Santa Barbara Island) and continued directional selection on Santa Cruz Island; additional studies to evaluate this possibility are warranted. Feral cats were present on both islands for a brief period from the late 1800s until they were largely eliminated by the 1950s (McChesney & Tershy, 1998). Although it is unknown whether mice on either island respond to olfactory cues of feral cats, it is clear that mice on Santa Barbara Island do not respond to cues from fox predators (Orrock, 2010). Our analysis was not able to fully analyze variation between the two islands in olfactory receptor genes (OLR). Given the expectation of about 900 functional olfactory receptors in most rodent species (Niimura & Nei, 2007) and current list of about 480 *Peromyscus* olfactory receptors, it is likely a large number of *Peromyscus* olfactory receptor genes exist but are missing from our analysis. Similarly, only 18 vomeronasal receptors are annotated for *Peromyscus*, and it is likely that hundreds are missing from our analysis and we cannot exclude that we are missing important changes in olfactory and vomeronasal receptors due to the lack of a complete annotated exome for *P. maniculatus*. Whole‐genome sequencing of individuals and careful discovery of predicted olfactory receptor gene sequences would allow one to overcome this obstacle.

Pathogens and parasites can have significant effects on host evolution (e.g., Boots et al., 2009). Our molecular results suggest that differences in pathogen prevalence and/or diversity between islands (Table 1) may have been selected for different genetic variants on different islands. As noted above, a remarkable 106 of the 709 outlier genes were related to immune function (Figure 2, Figure S3). Although extensive pathogen records do not exist for both islands, one striking difference is that mice on Santa Cruz Island have some of the highest rates of Sin Nombre virus (SNV) prevalence ever documented, while SNV has never been found in mice on Santa Barbara Island (Table 1; Graham & Chomel, 1997; Orrock et al., 2021). Molecular work with SNV demonstrates the long shared evolutionary history of mice and this virus on Santa Cruz, as SNV from Santa Cruz exhibits signs of divergence from mainland viral populations (Hjelle et al., 1996). Because Sin Nombre virus has been found to reduce the survival of reproductive adult *P. maniculatus*, especially males (Luis et al., 2012), we expect continued selection on the Santa Cruz Island mouse population but relaxed selection due to an absence of SNV in the Santa Barbara Island mouse population. Such relaxed selection on the Santa Barbara Island population may explain how this population has immune‐related genes exhibiting non‐synonymous mutations (in *Cyp19a1*, *Eif2ak4*, *Syne1*, three in *Tlr2*) and a missense mutation (in *Il12a*) that are not present in the Santa Cruz Island population, where high SNV prevalence would favor selection for functional copies of these genes.

We also discovered significant variation in genes that can influence both body size and pathogen resistance. For example, the Santa Cruz population has a fixed mutation of moderate effect in *Bc16*. Changes in *Bc16* are associated with reduced body size (Yoshida et al., 1999), and mice from Santa Cruz island exhibit longer tails than mice on Santa Barbara Island; studies from mainland *Peromyscus* suggest that longer tails (i.e., greater tail/body ratios) may be adaptive in settings with greater habitat complexity, such as forests compared with prairies (Kingsley et al., 2017). Santa Cruz Island has a larger number of habitat and vegetation types than Santa Barbara Island (Johnson & Rodriguez, 2001). *Bc16* is also associated with the ability to tolerate chronic viral infections (Harker et al., 2011), which might be advantageous on Santa Cruz Island where SNV prevalence is very high.

Densities of mice on Santa Barbara Island can be among the highest densities documented (Drost & Fellers, 1991; Schwemm et al., 2018); reduced aggression has been documented in deer mice on other islands where high densities are achieved (Halpin & Sullivan, 1978), but this change may have been due to social context and not heritable changes (Baier & Hoekstra, 2019). We observe reduced wounding (Table 1), despite very high densities (Schwemm et al., 2018), in the Santa Barbara island population, consistent with evolution of reduced aggression behavior. We found a moderate‐effect mutation in *Cyp19a*, the gene for aromatase that converts testosterone to estradiol, on Santa Barbara Island that could be associated with reduced aggression (Toda et al., 2001). *Lama2* mutations, which are also more common and predicted to have moderate effect on Santa Barbara Island, have also been found to affect aggression (Zhang‐James et al., 2019).

Given that these populations have been separated for quite some time, the potential exists for other forces (e.g., genetic drift due to founder effects, cyclic population dynamics) to contribute to genomic differences between islands. Such demographic changes may sometimes produce genomic signatures similar to those produced by selection, although demographic changes are expected to exert genome‐wide influence rather than acting on specific outliers. We did not estimate demographic parameters such as N_E_, given that population sizes may have changed many times in the last several thousand years, resulting in complicated demographic histories. We believe that our results were not substantially confounded by demography because we used methods that account for population structure and used multiple approaches to identify the strongest outliers. While we cannot rule out demographic history in affecting the signatures of selection we detected, our identification of outlier variants among genes associated with known phenotypic differences and non‐synonymous SNPs of potentially moderate to large effect suggests that selection may have acted on many of these genes. Our results set the stage for important future studies that examine the degree to which these documented phenotypic and genotypic differences among island populations lead to changes in fitness.

We used the STRING tool to further analyze the outlier genes because it provides information on interactions between genes and it is possible that highly interactive genes could synergize to have an oversized effect on phenotype. With STRING, we found a subset of 92 highly connected genes that are relevant to immune and nervous system processes (Figure 2). The gene with the highest interaction with other genes was *Itpkb*, an IP3 kinase. *Itpkb* plays an important role in immune cell differentiation (Pouillon et al., 2013). *Itpkb* appears to be involved in some neurological disorders, such as Alzheimer's disorder (Stygelbout et al., 2014), so exome alterations in this gene could have both immunological and neurobiological consequences. Here, we considered genes with identical names and symbol in both mice and humans to be true orthologs. Other top interactive genes included *App*, which plays a role in normal CNS function and is part of the Alzheimer's disorder pathway (Hardy, 2017). *Brca1* is involved in DNA repair and is linked to cancers, especially breast cancer (Zhu et al., 2016). *Atm* also has a role in DNA repair (Blackford & Jackson, 2017), while *Egf* codes for a growth factor that plays a role in neuronal differentiation (Garcez et al., 2009) and immune response (Kim et al., 2018). *Nr3c1*, the glucocorticoid (cortisol) receptor, connected with 8 other genes in this group (Figure 2) and is of interest because it has been well studied in the context of evolutionary pressure changes that affect both immune‐ and stress‐related responses in vertebrates (Stolte et al., 2006). It is possible that this receptor could be a key driver of some of the immunological and behavioral changes among island populations, but this hypothesis also requires further confirmation.

In demonstrating how exome capture and sequencing can inform the link between phenotypic variation and genotypic variation between island populations with contrasting selection pressures, our results highlight profitable future directions for exploring widespread patterns in phenotypic and molecular evolution. In further elucidating the molecular and phenotypic differences among island populations, our work sets the stage for future studies that may help us understand the limits to selection in island populations, as these populations of (often endemic) species are likely to be among the most threatened by global climate change (Courchamp et al., 2014). Our results also have implications beyond island systems. For example, understanding how animals respond to novel predators, pathogens, and environments is a primary focus of contemporary evolutionary biology. *Peromyscus maniculatus* has a geographic range that encompasses most of North America, making it one of the few mammalian species (other than humans) that experiences such a wide array of selective pressures. By helping to identify the source of molecular variation underlying behavioral, physiological, and morphological phenotypes of *P. maniculatus* on islands that differ in predators, pathogens, and population density, we hope to motivate studies that leverage the expansive geographic range of *P. maniculatus*, the extensive molecular tools available for this genus, and a growing literature on variation and adaptation in this genus (Baier & Hoekstra, 2019; Bedford & Hoekstra, 2015; Harris & Munshi‐South, 2017; Kingsley et al., 2017; Storz et al., 2012) to understand how a single species exhibits adaptive evolution across a wide array of environments.

## CONFLICT OF INTEREST

The authors declare that we have no conflict of interest.

## AUTHOR CONTRIBUTIONS

**John L. Orrock:** Conceptualization (lead); Formal analysis (supporting); Funding acquisition (lead); Investigation (equal); Project administration (lead); Visualization (equal); Writing‐original draft (lead). **Linelle Abueg:** Data curation (equal); Formal analysis (lead); Investigation (supporting); Methodology (equal); Visualization (supporting); Writing‐review & editing (equal). **Stephen Gammie:** Formal analysis (equal); Investigation (supporting); Methodology (equal); Visualization (equal); Writing‐review & editing (equal). **Jason Munshi‐South:** Conceptualization (equal); Formal analysis (equal); Funding acquisition (equal); Investigation (supporting); Methodology (lead); Project administration (supporting); Writing‐review & editing (equal).

## Supporting information

Appendix S1

## Data Availability

Raw FASTQ files for each individual used in this study have been archived on the NCBI SRA database and are freely available (project PRJNA775971, accession numbers SRR16611434‐SRR1661148).
