# Supplementary material for: Exome sequencing of deer mice on two California Channel Islands identifies potential adaptation to strongly contrasting ecological conditions
Source: Ecol Evol. 2021 Nov 17;11(23):17191–201. doi: 10.1002/ece3.8357 (PMC8668806; doi:10.1002/ece3.8357)
Supplement: Supplementary file 1 — Appendix S1 [file ECE3-11-17191-s001.docx]

**Appendix for: Exome sequencing of deer mice on two California Channel Islands identifies potential adaptation to strongly contrasting ecological conditions.**

John L. Orrock^1^; Linelle Abueg^2^; Stephen Gammie^1^; Jason Munshi-South^2^

^1^Department of Integrative Biology, University of Wisconsin, Madison, WI, USA

^2^Louis Calder Center – Biological Field Station, Fordham University, Armonk, NY, USA

**Appendix**


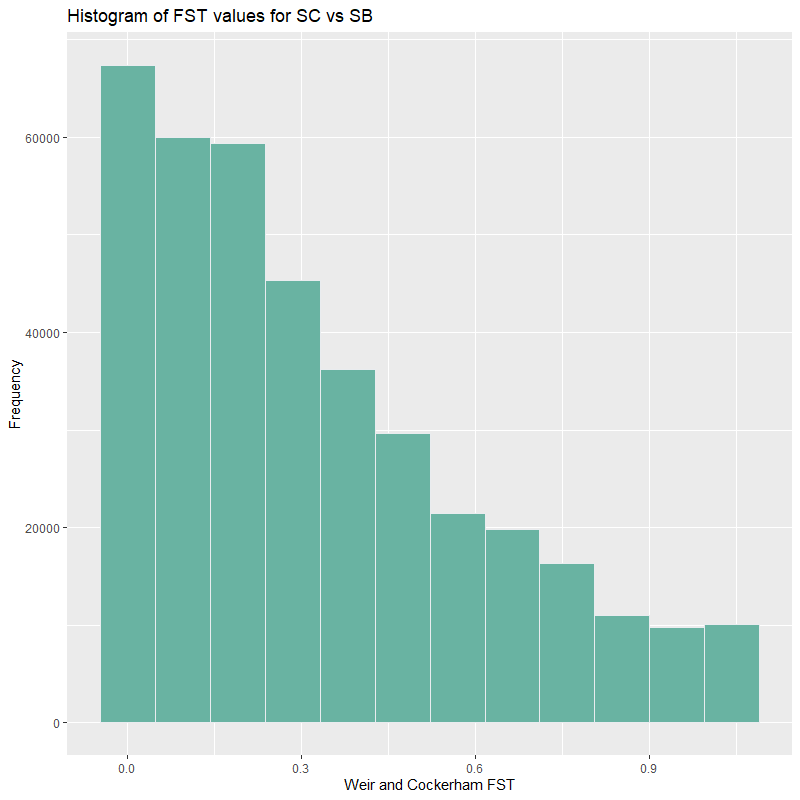
Appendix Fig. S1. Top panel: Density plot of FST values (top) from VCFTools (mean FST: 0.320, weighted FST: 0.435). Bottom panel: FST outlier plot from Bayescan (bottom).


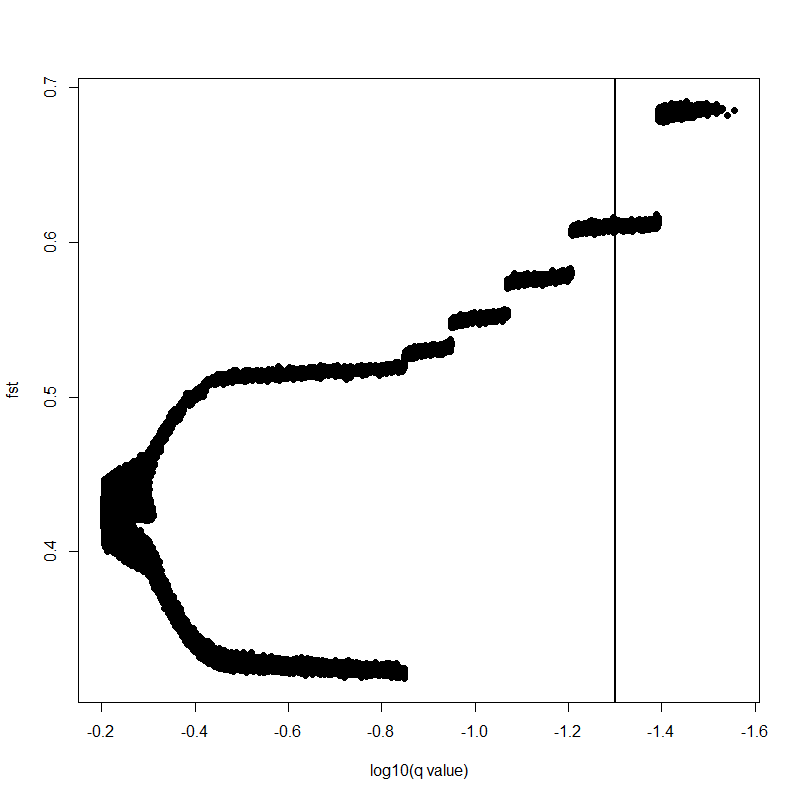


Appendix Fig. S2. Figure demonstrating almost complete genetic separation. Admixture coefficients for Santa Cruz and Santa Barbara mice given two ancestor groups. Individuals show distinct clustering according to island of origin.


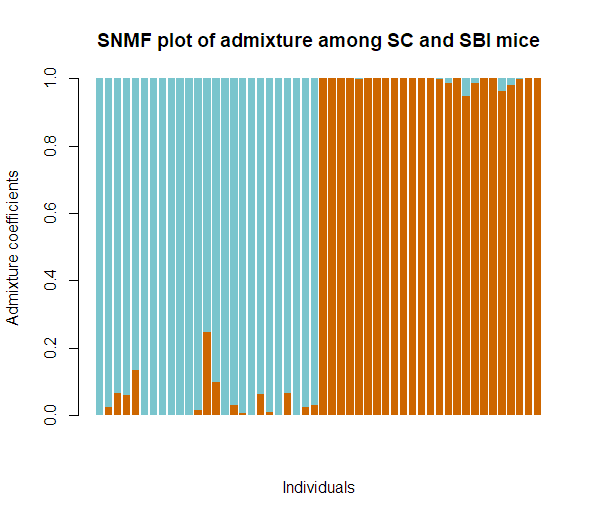


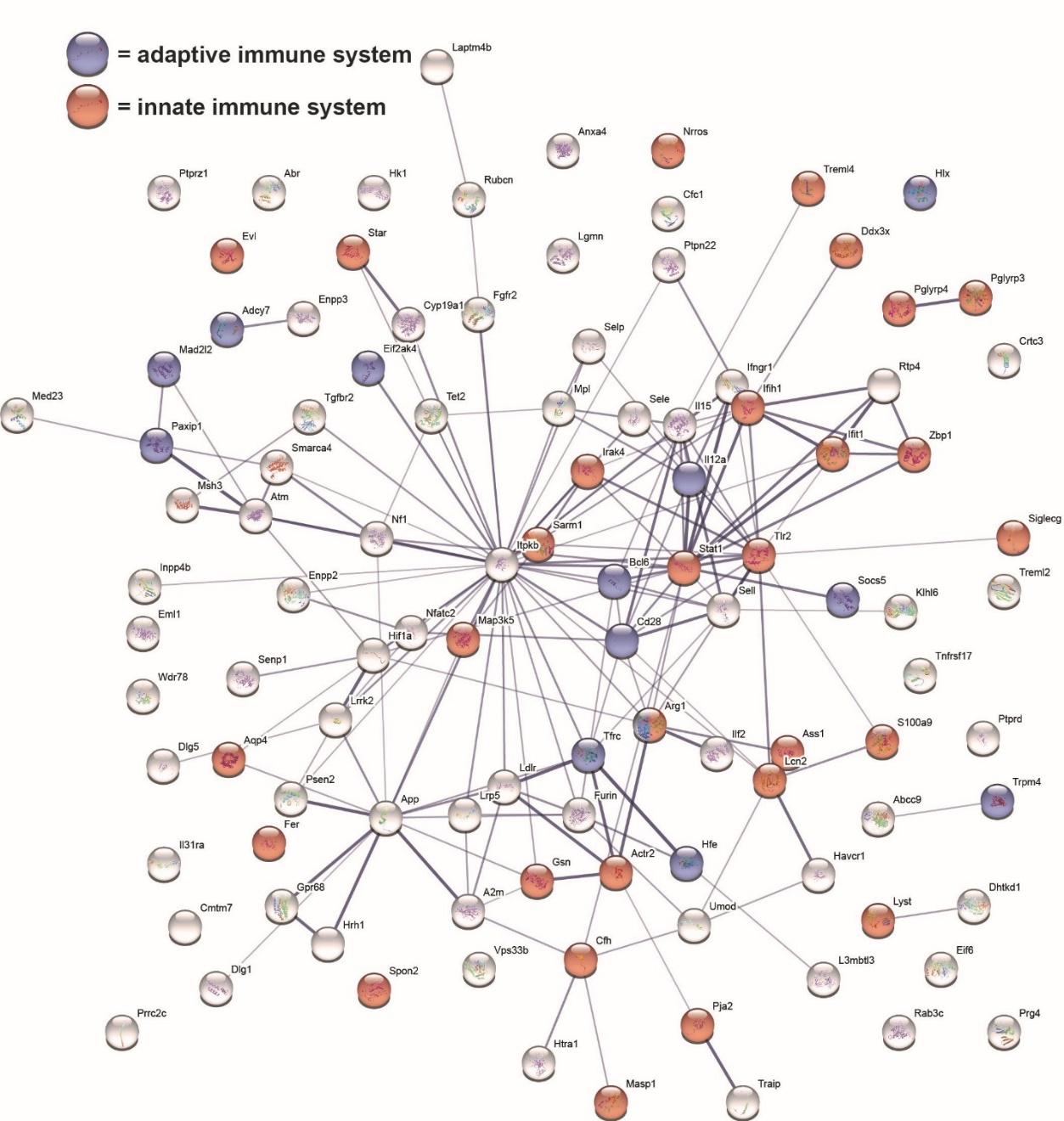
Appendix Figure S3. Plotting of immune-related genes. Using enrichment analysis of 709 outlier genes, STRING identified 105 as being related in some way to immune function. *Itpkb* was also plotted as the STRING enrichment did not recognize this gene and it has been implicated in immune response (see above). Circles are genes and lines (edges) between genes indicates and known connection. Circles are colored according to known function for a subset, including innate immune system (red) and adaptive immune system (blue). Color of line indicates type of evidence (details provided within STRING database).

Appendix Table S1. Genes that contained outlier SNPs identified by BayeScan, pcadapt, and LFMM.

| Gene.symbol |
| --- |
| A2m |
| Aaed1 |
| Abca3 |
| Abcc12 |
| Abcc9 |
| Abi3bp |
| Ablim3 |
| Abr |
| Acap2 |
| Aco1 |
| Acot6 |
| Acsm1 |
| Acsm3 |
| Acsm4 |
| Acta2 |
| Actr2 |
| Adam29 |
| Adamts20 |
| Adat1 |
| Adck3 |
| Adcy7 |
| Adgrg2 |
| Adgrv1 |
| Adh4 |
| Adh6b |
| Adh7 |
| Afmid |
| Aftph |
| Ago4 |
| Ak4 |
| Akap12 |
| Akr1c13 |
| Akr1c14 |
| Akr1c18 |
| Alad |
| Aldh16a1 |
| Aldh7a1 |
| Alg9 |
| Ambp |
| Amy2a1 |
| Ankrd1 |
| Ankrd13b |
| Ankrd22 |
| Ankrd34b |
| Ankrd44 |
| Anks1b |
| Ano4 |
| Ano8 |
| Anxa4 |
| Aox2 |
| Ap2m1 |
| Apcdd1l |
| App |
| Aqp4 |
| Arap2 |
| Arg1 |
| Arhgap18 |
| Arhgap20 |
| Arhgap26 |
| Arhgap32 |
| Arhgef17 |
| Arhgef4 |
| Arid4a |
| Armt1 |
| Arrdc4 |
| Asb5 |
| Asb8 |
| Ascc2 |
| Ash2l |
| Aspg |
| Asprv1 |
| Ass1 |
| Ate1 |
| Atf7ip |
| Atg16l1 |
| Atm |
| Atp10a |
| Atp13a5 |
| Atp4b |
| Atp8b3 |
| Atxn2 |
| Bace1 |
| Bcl6 |
| Brca1 |
| Brd3 |
| Bre |
| Brinp3 |
| Bub1b |
| C2cd2 |
| C2cd5 |
| Cacna2d1 |
| Calm1 |
| Caln1 |
| Capn8 |
| Cars2 |
| Casc5 |
| Cask |
| Ccdc121 |
| Ccdc150 |
| Ccdc154 |
| Ccdc170 |
| Ccdc57 |
| Ccdc67 |
| Ccdc88c |
| Ccndbp1 |
| Cd28 |
| Cdc42bpg |
| Cdhr4 |
| Cdk15 |
| Cdrt1 |
| Ceacam18 |
| Cenpl |
| Cep164 |
| Cep295 |
| Cfap46 |
| Cfap52 |
| Cfap57 |
| Cfc1 |
| Cfh |
| Chd1 |
| Chd8 |
| Chfr |
| Chil3 |
| Chil6 |
| Chl1 |
| Cilp |
| Clcn3 |
| Clcn7 |
| Clip2 |
| Clk1 |
| Clstn2 |
| Cltc |
| Cmas |
| Cmtm7 |
| Cmtm8 |
| Cnbd1 |
| Cnksr3 |
| Cntn4 |
| Col15a1 |
| Col4a3 |
| Col6a4 |
| Col7a1 |
| Colca2 |
| Colec10 |
| Cpn2 |
| Crfhr |
| Crtap |
| Crtc3 |
| Cry1 |
| Crybg3 |
| Csmd2 |
| Ctcfl |
| Cyp19a1 |
| Cyp4a10 |
| Cyp4f16 |
| Dars2 |
| Dcc |
| Dclk2 |
| Ddx20 |
| Ddx3x |
| Ddx4 |
| Dennd4a |
| Dgke |
| Dhfr |
| Dhtkd1 |
| Dhx29 |
| Dip2b |
| Disp3 |
| Dkk2 |
| Dlg1 |
| Dlg5 |
| Dnah14 |
| Dnah17 |
| Dnah5 |
| Dnajc13 |
| Dnajc8 |
| Dnal1 |
| Dnmt3l |
| Dock6 |
| Dock9 |
| Dpp8 |
| Dscam |
| Dscaml1 |
| Dynap |
| Dynlt3 |
| Eaf2 |
| Ebf2 |
| Efcab11 |
| Efcab5 |
| Efl1 |
| Egf |
| Eif2ak4 |
| Eif4g1 |
| Eif6 |
| Elavl3 |
| Emcn |
| Eml1 |
| Eml5 |
| Enah |
| Eno1 |
| Enoph1 |
| Enpp2 |
| Enpp3 |
| Epor |
| Ercc6l |
| Ercc6l2 |
| Eri2 |
| Erp27 |
| Eva1c |
| Evc |
| Evc2 |
| Evl |
| Evpl |
| Exoc7 |
| Exph5 |
| Eya3 |
| Fam114a2 |
| Fam124b |
| Fam126b |
| Fam135a |
| Fam186a |
| Fam193a |
| Fam49a |
| Fat3 |
| Fbrsl1 |
| Fcgbp |
| Fer |
| Fermt1 |
| Fgd6 |
| Fgfr2 |
| Fggy |
| Fmo1 |
| Fmo2 |
| Fmo6 |
| Fndc3a |
| Fnta |
| Frem3 |
| Frmpd1 |
| Ftsj1 |
| Furin |
| Fxyd2 |
| Fyttd1 |
| Gab1 |
| Gabarapl2 |
| Gabra5 |
| Gabrg3 |
| Gabrr3 |
| Gatad2b |
| Gdpd1 |
| Gemin5 |
| Gene |
| Gfpt1 |
| Gfy |
| Gga1 |
| Ggt6 |
| Ggta1 |
| Gif |
| Gjb5 |
| Glb1 |
| Gls |
| Gmcl1 |
| Gnptab |
| Gpc4 |
| Gpr68 |
| Greb1 |
| Grid2ip |
| Grik1 |
| Grxcr1 |
| Gsn |
| Gtf2i |
| Gtf3c3 |
| Gucy2c |
| Gzmk |
| Hacd3 |
| Havcr1 |
| Hdac10 |
| Herc3 |
| Hfe |
| Hgsnat |
| Hif1a |
| Hk1 |
| Hlx |
| Hmcn1 |
| Hnrnpd |
| Hook1 |
| Hook3 |
| Hpgd |
| Hrasls |
| Hrh1 |
| Htr7 |
| Htra1 |
| Hunk |
| Hyi |
| Icoslg |
| Ifih1 |
| Ifit1 |
| Ifngr1 |
| Ift122 |
| Ift80 |
| Igdcc4 |
| Ikbkap |
| Il12a |
| Il15 |
| Il19 |
| Il20ra |
| Il31ra |
| Ildr1 |
| Ilf2 |
| Immt |
| Inpp4b |
| Ints3 |
| Ip6k1 |
| Ipo11 |
| Iqck |
| Irak4 |
| Itga8 |
| Itpka |
| Itpkb |
| Kalrn |
| Kat6b |
| Kcnd3 |
| Kcnj1 |
| Kcnk10 |
| Kcnq1 |
| Kcns3 |
| Kcnu1 |
| Kctd12b |
| Kdelc2 |
| Kdm1b |
| Kdm3a |
| Kdm4a |
| Kel |
| Kiaa0753 |
| Kif2a |
| Kif5c |
| Kiz |
| Klhl6 |
| Kmt2d |
| Kndc1 |
| Kntc1 |
| Krt71 |
| Krt74 |
| Krt78 |
| L3mbtl3 |
| Lama1 |
| Lama2 |
| Laptm4b |
| Larp7 |
| Lbr |
| Lcn2 |
| Ldlr |
| Lgals3bp |
| Lgmn |
| Lima1 |
| Lipa |
| Liph |
| Lipm |
| Lmln |
| Lmo7 |
| Lonp1 |
| Loxhd1 |
| Lrba |
| Lrch3 |
| Lrig2 |
| Lrit3 |
| Lrp10 |
| Lrp2 |
| Lrp5 |
| Lrrk2 |
| Lsm1 |
| Ltn1 |
| Lyst |
| Mad2l2 |
| Madd |
| Man1a1 |
| Man2b2 |
| Map1a |
| Map3k4 |
| Map3k5 |
| Map4k1 |
| Map7 |
| Mapk11 |
| Mapk12 |
| Masp1 |
| Mbd2 |
| Mcf2l |
| Mcm3 |
| Mdm4 |
| Mdn1 |
| Med23 |
| Med8 |
| Mgat4d |
| Mgat5b |
| Mmp24 |
| Mpl |
| Mroh9 |
| Mrpl22 |
| Mrpl45 |
| Msh3 |
| Mta1 |
| Mterf2 |
| Mtfr2 |
| Mtmr3 |
| Mttp |
| Muc20 |
| Muc4 |
| Mxra7 |
| Myo3a |
| Myocd |
| Myom1 |
| Myot |
| Nacc1 |
| Nbr1 |
| Ncbp1 |
| Ndufaf2 |
| Ndufb3 |
| Nedd9 |
| Nell2 |
| Nes |
| Nf1 |
| Nfatc2 |
| Nfs1 |
| Nhsl2 |
| Nid2 |
| Nipal1 |
| Nln |
| Nmd3 |
| Npepl1 |
| Npr1 |
| Nr3c1 |
| Nrde2 |
| Nrip1 |
| Nrros |
| Nsd1 |
| Ntf3 |
| Nuak1 |
| Nucb1 |
| Nudt5 |
| Nup153 |
| Oca2 |
| Olfr1340 |
| Olfr1420 |
| Olfr1431 |
| Olfr1434 |
| Olfr1440 |
| Olfr177 |
| Olfr196 |
| Olfr206 |
| Olfr215 |
| Olfr727 |
| Olfr76 |
| Orc1 |
| Orc2 |
| Ovgp1 |
| Pak2 |
| Pank1 |
| Pappa2 |
| Pard3 |
| Pard3b |
| Parp16 |
| Patl1 |
| Paxip1 |
| Pck1 |
| Pcyox1 |
| Pdcd7 |
| Pde4b |
| Pepd |
| Pex7 |
| Pfas |
| Pfkfb4 |
| Pfkm |
| Pgap1 |
| Pglyrp3 |
| Pglyrp4 |
| Phc2 |
| Pi4kb |
| Pigs |
| Pik3c2b |
| Pik3c2g |
| Pik3r5 |
| Pitrm1 |
| Pja2 |
| Pkd1 |
| Pkd1l1 |
| Pkhd1 |
| Pla2g4f |
| Plbd1 |
| Plce1 |
| Plcl1 |
| Plekha5 |
| Plxdc2 |
| Plxnb2 |
| Pole3 |
| Poln |
| Polr1a |
| Pou2f3 |
| Ppil3 |
| Ppip5k1 |
| Ppp1r15a |
| Ppp1r16a |
| Ppp4r3a |
| Prep |
| Prg4 |
| Prkag1 |
| Prrc2c |
| Prss34 |
| Psen2 |
| Psma3 |
| Psmd2 |
| Psrc1 |
| Ptcd3 |
| Ptpn18 |
| Ptpn22 |
| Ptprd |
| Ptprz1 |
| Rab3c |
| Rab3d |
| Rangap1 |
| Rasgrp3 |
| Rasgrp4 |
| Rasip1 |
| Raver2 |
| Rbm34 |
| Rbm39 |
| Rerg |
| Rfwd2 |
| Rfx4 |
| Rgl3 |
| Rgmb |
| Rgs6 |
| Rin3 |
| Rmdn2 |
| Rmdn3 |
| Rnase4 |
| Rnf123 |
| Rnf214 |
| Robo2 |
| Rpp30 |
| Rps4x |
| Rps6kc1 |
| Rtn2 |
| Rtp4 |
| Rubcn |
| Runx1t1 |
| Ryr1 |
| S100a4 |
| S100a5 |
| S100a9 |
| Sarm1 |
| Sars2 |
| Scaf8 |
| Sco1 |
| Sec31a |
| Sec61a2 |
| Sel1l |
| Sele |
| Sell |
| Selp |
| Senp1 |
| Sesn2 |
| Sf3a3 |
| Sgcz |
| Sgip1 |
| Shc1 |
| Siglecg |
| Sipa1l1 |
| Slc13a5 |
| Slc15a2 |
| Slc16a12 |
| Slc1a4 |
| Slc23a3 |
| Slc24a1 |
| Slc25a11 |
| Slc35d1 |
| Slc36a4 |
| Slc38a9 |
| Slc51b |
| Slc5a1 |
| Slc6a17 |
| Slitrk6 |
| Smarca4 |
| Smarcal1 |
| Smurf1 |
| Snapin |
| Snx30 |
| Socs5 |
| Spag1 |
| Spata31d1c |
| Spon2 |
| Srp68 |
| Ssh2 |
| St8sia1 |
| Stab2 |
| Stambpl1 |
| Star |
| Stat1 |
| Stat4 |
| Stk32a |
| Stmn4 |
| Stom |
| Stradb |
| Strip1 |
| Stx10 |
| Stx3 |
| Sult6b1 |
| Sync |
| Syne1 |
| Syngr2 |
| Syt1 |
| Szt2 |
| Tacc2 |
| Taf1d |
| Tbc1d9 |
| Tcf20 |
| Tctex1d1 |
| Tecpr2 |
| Ten1 |
| Tet2 |
| Tex11 |
| Tfrc |
| Tgfbr2 |
| Thumpd1 |
| Tiam1 |
| Tk1 |
| Tlr2 |
| Tm4sf20 |
| Tmem106a |
| Tmem106c |
| Tmem114 |
| Tmem117 |
| Tmem132c |
| Tmem150c |
| Tmem245 |
| Tmem45a2 |
| Tmem87a |
| Tnfrsf17 |
| Tnn |
| Tnr |
| Tns1 |
| Tp53bp1 |
| Tpcn2 |
| Tpmt |
| Tpr |
| Traf3ip2 |
| Traf7 |
| Traip |
| Trak2 |
| Treml2 |
| Treml4 |
| Trim16 |
| Trio |
| Trmt1 |
| Trmt10a |
| Trpm4 |
| Tsc2 |
| Tsr3 |
| Ttc8 |
| Ttll13 |
| Tubd1 |
| Tubgcp4 |
| Tubgcp6 |
| Tvp23b |
| Uba7 |
| Ubr3 |
| Ucn2 |
| Ugt1a6a |
| Umod |
| Unc5a |
| Unc5d |
| Upk2 |
| Urb1 |
| Usp43 |
| Utrn |
| Vasp |
| Vmn1r32 |
| Vps33b |
| Vps39 |
| Vps8 |
| Vwa5a |
| Vwa9 |
| Wbp11 |
| Wdfy3 |
| Wdfy4 |
| Wdr5 |
| Wdr78 |
| Wfs1 |
| Wrn |
| Xaf1 |
| Xdh |
| Xpa |
| Zbp1 |
| Zc3h12c |
| Zcchc11 |
| Zcchc8 |
| Zdhhc4 |
| Zfand4 |
| Zfp658 |
| Zfp719 |
| Zfpm2 |
| Zfyve28 |
| Zgrf1 |
| Zmat4 |
| Znf236 |
| Znf239 |
| Znf250 |
| Znf346 |
| Znf853 |
| Zscan20 |
| Zyg11b |

Appendix Table S2. Additional information regarding 19 mutations in SNPs within the 35 candidate genes where frequencies were not completely fixed within an island population. The remaining 124 mutations within the 35 candidate genes were completely fixed with an island population.

| Gene | Predicted variant effect | Number of SNP variants | Variant frequency | |
| --- | --- | --- | --- | --- |
|  |  |  | Santa Barbara Island | Santa Cruz Island |
| *Olfr727* | Low | 1 | 0 | 0.954 |
| *Tlr2* | Low | 3 | 1 | 0.045 |
| *Tlr2* | Low | 2 | 0 | 0.954 |
| *Tlr2* | Moderate | 3 | 1 | 0.045 |
| *App* | Low | 1 | 0 | 0.954 |
| *Cyp19a1* | Low | 1 | 0.98 | 0 |
| *Bcl6* | Low | 1 | 0.98 | 0 |
| *Eif2ak4* | Low | 1 | 0 | 0.977 |
| *Eif2ak4* | Low | 1 | 1 | 0.023 |
| *Dcc* | Low | 1 | 1 | 0.045 |
| *Lama2* | Low | 1 | 1 | 0.023 |
| *Ryr1* | Low | 2 | 1 | 0.045 |
| *Ryr1* | Low | 1 | 0 | 0.954 |
| *App* | Modifier | 1 | 0 | 0.98 |
| *Bc16* | Modifier | 1 | 0 | 0.98 |
| *Eif2ak4* | Modifier | 4 | 1 | 0.023 |
| *Stat1* | Modifier | 1 | 0 | 0.96 |
| *Stat1* | Modifier | 5 | 1 | 0.045 |
| *Lama2* | Modifier | 5 | 1 | 0.045 |
| *Lama2* | Modifier | 1 | 0 | 0.96 |
| *Lama2* | Modifier | 1 | 0 | 0.98 |
| *Ryr1* | Modifier | 2 | 1 | 0.023 |
| *Ryr1* | Modifier | 1 | 1 | 0.045 |
| *Ryr1* | Modifier | 1 | 0.04 | 1 |
| *Syne1* | Modifier | 1 | 0 | 0.96 |
| *Syne1* | Modifier | 1 | 0 | 0.98 |
| *Syne1* | Modifier | 1 | 0 | 0.98 |
| *Syne1* | Modifier | 1 | 1 | 0.045 |

Appendix Table S3. PANTHER version 16 GO biological process overrepresentation results for the 709 genes identified by outlier analysis (Appendix Table S1).

| Analysis Type: | PANTHER Overrepresentation Test (Released 20210224) | | | | |
| --- | --- | --- | --- | --- | --- |
| Annotation Version and Release Date: | GO Ontology database DOI: 10.5281/zenodo.5228828 Released 2021-08-18 | | | | |
| Analyzed List: | Client Text Box Input (Mus musculus) | | | | |
| Reference List: | Mus musculus (all genes in database) | | | | |
| Test Type: | FISHER | | | | |
| Correction: | FDR | | | | |
| GO biological process complete | Mus musculus - REFLIST (21988) | Client Text Box Input (712) | Client Text Box Input (fold Enrichment) | Client Text Box Input (raw P-value) | Client Text Box Input (FDR) |
| renal absorption (GO:0070293) | 20 | 6 | 9.26 | 1.25E-04 | 2.75E-02 |
| phosphatidylinositol phosphate biosynthetic process (GO:0046854) | 46 | 8 | 5.37 | 2.62E-04 | 4.43E-02 |
| negative regulation of immune effector process (GO:0002698) | 129 | 14 | 3.35 | 1.68E-04 | 3.39E-02 |
| phosphatidylinositol metabolic process (GO:0046488) | 137 | 14 | 3.16 | 2.96E-04 | 4.86E-02 |
| cell-cell adhesion via plasma-membrane adhesion molecules (GO:0098742) | 193 | 19 | 3.04 | 4.24E-05 | 1.42E-02 |
| negative regulation of cellular catabolic process (GO:0031330) | 240 | 23 | 2.96 | 1.03E-05 | 4.91E-03 |
| axon guidance (GO:0007411) | 239 | 22 | 2.84 | 2.81E-05 | 1.03E-02 |
| neuron projection guidance (GO:0097485) | 240 | 22 | 2.83 | 2.98E-05 | 1.07E-02 |
| negative regulation of catabolic process (GO:0009895) | 306 | 26 | 2.62 | 1.99E-05 | 7.63E-03 |
| axonogenesis (GO:0007409) | 360 | 28 | 2.4 | 4.61E-05 | 1.45E-02 |
| renal system development (GO:0072001) | 319 | 24 | 2.32 | 2.48E-04 | 4.30E-02 |
| axon development (GO:0061564) | 392 | 29 | 2.28 | 9.09E-05 | 2.27E-02 |
| cell-cell adhesion (GO:0098609) | 422 | 30 | 2.2 | 1.66E-04 | 3.39E-02 |
| chemotaxis (GO:0006935) | 500 | 34 | 2.1 | 1.20E-04 | 2.67E-02 |
| taxis (GO:0042330) | 505 | 34 | 2.08 | 1.32E-04 | 2.82E-02 |
| cell adhesion (GO:0007155) | 863 | 58 | 2.08 | 5.45E-07 | 3.73E-04 |
| phosphorylation (GO:0016310) | 825 | 55 | 2.06 | 1.45E-06 | 9.13E-04 |
| biological adhesion (GO:0022610) | 873 | 58 | 2.05 | 6.79E-07 | 4.46E-04 |
| microtubule cytoskeleton organization (GO:0000226) | 521 | 34 | 2.02 | 2.74E-04 | 4.60E-02 |
| intracellular signal transduction (GO:0035556) | 1350 | 88 | 2.01 | 1.61E-09 | 4.23E-06 |
| microtubule-based process (GO:0007017) | 785 | 51 | 2.01 | 6.39E-06 | 3.25E-03 |
| protein phosphorylation (GO:0006468) | 656 | 42 | 1.98 | 5.81E-05 | 1.64E-02 |
| cell morphogenesis involved in differentiation (GO:0000904) | 586 | 37 | 1.95 | 2.44E-04 | 4.32E-02 |
| cell morphogenesis (GO:0000902) | 757 | 46 | 1.88 | 1.12E-04 | 2.55E-02 |
| carboxylic acid metabolic process (GO:0019752) | 787 | 47 | 1.84 | 1.05E-04 | 2.44E-02 |
| small molecule metabolic process (GO:0044281) | 1458 | 87 | 1.84 | 9.22E-08 | 1.12E-04 |
| phosphate-containing compound metabolic process (GO:0006796) | 1596 | 95 | 1.84 | 2.54E-08 | 3.34E-05 |
| phosphorus metabolic process (GO:0006793) | 1613 | 96 | 1.84 | 2.02E-08 | 3.18E-05 |
| organophosphate metabolic process (GO:0019637) | 757 | 45 | 1.84 | 1.76E-04 | 3.50E-02 |
| organic acid metabolic process (GO:0006082) | 830 | 49 | 1.82 | 1.02E-04 | 2.44E-02 |
| oxoacid metabolic process (GO:0043436) | 803 | 47 | 1.81 | 1.79E-04 | 3.52E-02 |
| carbohydrate derivative metabolic process (GO:1901135) | 828 | 48 | 1.79 | 2.17E-04 | 3.98E-02 |
| cellular lipid metabolic process (GO:0044255) | 885 | 51 | 1.78 | 1.60E-04 | 3.32E-02 |
| lipid metabolic process (GO:0006629) | 1135 | 65 | 1.77 | 1.77E-05 | 7.16E-03 |
| cell projection organization (GO:0030030) | 1190 | 68 | 1.76 | 1.25E-05 | 5.48E-03 |
| regulation of catabolic process (GO:0009894) | 902 | 51 | 1.75 | 1.96E-04 | 3.71E-02 |
| cytoskeleton organization (GO:0007010) | 1126 | 63 | 1.73 | 5.07E-05 | 1.48E-02 |
| plasma membrane bounded cell projection organization (GO:0120036) | 1134 | 63 | 1.72 | 5.57E-05 | 1.60E-02 |
| positive regulation of cellular component organization (GO:0051130) | 1237 | 65 | 1.62 | 2.11E-04 | 3.90E-02 |
| movement of cell or subcellular component (GO:0006928) | 1487 | 78 | 1.62 | 4.54E-05 | 1.46E-02 |
| macromolecule modification (GO:0043412) | 2449 | 126 | 1.59 | 3.82E-07 | 3.01E-04 |
| positive regulation of cell communication (GO:0010647) | 1731 | 88 | 1.57 | 4.36E-05 | 1.43E-02 |
| establishment of localization in cell (GO:0051649) | 1577 | 80 | 1.57 | 1.28E-04 | 2.77E-02 |
| positive regulation of signaling (GO:0023056) | 1738 | 88 | 1.56 | 5.95E-05 | 1.65E-02 |
| cellular protein modification process (GO:0006464) | 2272 | 114 | 1.55 | 5.15E-06 | 2.80E-03 |
| protein modification process (GO:0036211) | 2272 | 114 | 1.55 | 5.15E-06 | 2.70E-03 |
| regulation of protein modification process (GO:0031399) | 1537 | 77 | 1.55 | 2.61E-04 | 4.47E-02 |
| negative regulation of response to stimulus (GO:0048585) | 1638 | 81 | 1.53 | 2.27E-04 | 4.07E-02 |
| organic substance biosynthetic process (GO:1901576) | 2093 | 102 | 1.51 | 5.96E-05 | 1.62E-02 |
| cellular biosynthetic process (GO:0044249) | 2019 | 98 | 1.5 | 1.04E-04 | 2.44E-02 |
| regulation of cellular component organization (GO:0051128) | 2498 | 121 | 1.5 | 1.23E-05 | 5.53E-03 |
| cellular protein metabolic process (GO:0044267) | 2917 | 141 | 1.49 | 1.90E-06 | 1.15E-03 |
| regulation of cell communication (GO:0010646) | 3270 | 158 | 1.49 | 3.81E-07 | 3.16E-04 |
| protein metabolic process (GO:0019538) | 3482 | 168 | 1.49 | 1.42E-07 | 1.40E-04 |
| regulation of signaling (GO:0023051) | 3280 | 158 | 1.49 | 4.00E-07 | 3.00E-04 |
| biosynthetic process (GO:0009058) | 2159 | 103 | 1.47 | 1.01E-04 | 2.45E-02 |
| cellular localization (GO:0051641) | 2267 | 108 | 1.47 | 8.26E-05 | 2.10E-02 |
| response to chemical (GO:0042221) | 3543 | 168 | 1.46 | 4.02E-07 | 2.88E-04 |
| regulation of cellular protein metabolic process (GO:0032268) | 2346 | 111 | 1.46 | 8.18E-05 | 2.11E-02 |
| regulation of protein metabolic process (GO:0051246) | 2522 | 119 | 1.46 | 5.00E-05 | 1.49E-02 |
| organelle organization (GO:0006996) | 3251 | 153 | 1.45 | 2.47E-06 | 1.44E-03 |
| nervous system development (GO:0007399) | 2114 | 99 | 1.45 | 3.08E-04 | 5.00E-02 |
| nucleobase-containing compound metabolic process (GO:0006139) | 2223 | 104 | 1.44 | 2.03E-04 | 3.80E-02 |
| primary metabolic process (GO:0044238) | 6405 | 299 | 1.44 | 6.98E-13 | 3.66E-09 |
| macromolecule localization (GO:0033036) | 2295 | 107 | 1.44 | 1.93E-04 | 3.75E-02 |
| response to external stimulus (GO:0009605) | 2425 | 113 | 1.44 | 1.32E-04 | 2.78E-02 |
| cellular macromolecule metabolic process (GO:0044260) | 4038 | 188 | 1.44 | 1.94E-07 | 1.80E-04 |
| organonitrogen compound metabolic process (GO:1901564) | 4391 | 203 | 1.43 | 9.71E-08 | 1.09E-04 |
| heterocycle metabolic process (GO:0046483) | 2344 | 108 | 1.42 | 2.81E-04 | 4.66E-02 |
| organic cyclic compound metabolic process (GO:1901360) | 2656 | 122 | 1.42 | 1.19E-04 | 2.67E-02 |
| regulation of localization (GO:0032879) | 2915 | 133 | 1.41 | 6.91E-05 | 1.84E-02 |
| regulation of molecular function (GO:0065009) | 2522 | 115 | 1.41 | 2.23E-04 | 4.04E-02 |
| cellular component organization (GO:0016043) | 5271 | 240 | 1.41 | 8.73E-09 | 1.53E-05 |
| cellular metabolic process (GO:0044237) | 6518 | 295 | 1.4 | 5.50E-11 | 1.73E-07 |
| organic substance metabolic process (GO:0071704) | 7141 | 323 | 1.4 | 2.34E-12 | 9.23E-09 |
| cellular component organization or biogenesis (GO:0071840) | 5463 | 247 | 1.4 | 7.92E-09 | 1.56E-05 |
| metabolic process (GO:0008152) | 7571 | 340 | 1.39 | 6.41E-13 | 5.05E-09 |
| macromolecule metabolic process (GO:0043170) | 5460 | 245 | 1.39 | 2.16E-08 | 3.10E-05 |
| negative regulation of cellular process (GO:0048523) | 4932 | 221 | 1.38 | 1.97E-07 | 1.73E-04 |
| regulation of multicellular organismal process (GO:0051239) | 2858 | 128 | 1.38 | 1.94E-04 | 3.74E-02 |
| nitrogen compound metabolic process (GO:0006807) | 5847 | 261 | 1.38 | 7.61E-09 | 1.71E-05 |
| negative regulation of biological process (GO:0048519) | 5380 | 238 | 1.37 | 1.36E-07 | 1.43E-04 |
| regulation of biological quality (GO:0065008) | 3876 | 168 | 1.34 | 8.08E-05 | 2.12E-02 |
| system development (GO:0048731) | 4223 | 180 | 1.32 | 9.27E-05 | 2.28E-02 |
| developmental process (GO:0032502) | 5627 | 239 | 1.31 | 3.09E-06 | 1.74E-03 |
| multicellular organism development (GO:0007275) | 4842 | 205 | 1.31 | 3.60E-05 | 1.23E-02 |
| anatomical structure development (GO:0048856) | 5233 | 220 | 1.3 | 2.20E-05 | 8.25E-03 |
| localization (GO:0051179) | 5066 | 212 | 1.29 | 4.79E-05 | 1.45E-02 |
| positive regulation of cellular process (GO:0048522) | 5825 | 241 | 1.28 | 1.97E-05 | 7.78E-03 |
| positive regulation of biological process (GO:0048518) | 6350 | 257 | 1.25 | 4.62E-05 | 1.43E-02 |
| cellular process (GO:0009987) | 15286 | 597 | 1.21 | 5.72E-18 | 9.01E-14 |
| regulation of cellular process (GO:0050794) | 11583 | 425 | 1.13 | 2.46E-04 | 4.30E-02 |
| biological_process (GO:0008150) | 20676 | 695 | 1.04 | 1.43E-05 | 5.94E-03 |
| G protein-coupled receptor signaling pathway (GO:0007186) | 1821 | 28 | 0.47 | 7.30E-06 | 3.59E-03 |
| Unclassified (UNCLASSIFIED) | 1312 | 17 | 0.4 | 1.43E-05 | 6.10E-03 |
| sensory perception of smell (GO:0007608) | 1123 | 14 | 0.38 | 3.51E-05 | 1.23E-02 |
| sensory perception of chemical stimulus (GO:0007606) | 1223 | 15 | 0.38 | 1.09E-05 | 5.07E-03 |

Appendix Table S4. PANTHER version 16 GO molecular function overrepresentation results for the 709 genes identified by outlier analysis (Appendix Table S1).

| Analysis Type: | PANTHER Overrepresentation Test (Released 20210224) | | | | |
| --- | --- | --- | --- | --- | --- |
| Annotation Version and Release Date: | GO Ontology database DOI: 10.5281/zenodo.5228828 Released 2021-08-18 | | | | |
| Analyzed List: | Client Text Box Input (Mus musculus) | | | | |
| Reference List: | Mus musculus (all genes in database) | | | | |
| Test Type: | FISHER | | | | |
| Correction: | FDR | | | | |
| GO molecular function complete | Mus musculus - REFLIST (21988) | Client Text Box Input (712) | Client Text Box Input (fold Enrichment) | Client Text Box Input (raw P-value) | Client Text Box Input (FDR) |
| inositol hexakisphosphate kinase activity (GO:0000828) | 8 | 4 | 15.44 | 3.88E-04 | 4.78E-02 |
| guanyl-nucleotide exchange factor activity (GO:0005085) | 197 | 21 | 3.29 | 5.63E-06 | 1.12E-03 |
| GTPase activator activity (GO:0005096) | 405 | 34 | 2.59 | 2.14E-06 | 5.12E-04 |
| GTPase regulator activity (GO:0030695) | 421 | 35 | 2.57 | 1.63E-06 | 4.60E-04 |
| nucleoside-triphosphatase regulator activity (GO:0060589) | 421 | 35 | 2.57 | 1.63E-06 | 4.35E-04 |
| microtubule binding (GO:0008017) | 266 | 21 | 2.44 | 4.09E-04 | 4.90E-02 |
| tubulin binding (GO:0015631) | 380 | 29 | 2.36 | 4.46E-05 | 7.37E-03 |
| ATP-dependent activity (GO:0140657) | 539 | 36 | 2.06 | 8.57E-05 | 1.37E-02 |
| kinase activity (GO:0016301) | 735 | 48 | 2.02 | 1.37E-05 | 2.53E-03 |
| phosphotransferase activity, alcohol group as acceptor (GO:0016773) | 680 | 44 | 2 | 3.02E-05 | 5.18E-03 |
| ATP binding (GO:0005524) | 1397 | 89 | 1.97 | 4.60E-09 | 5.51E-06 |
| transferase activity, transferring phosphorus-containing groups (GO:0016772) | 875 | 54 | 1.91 | 1.28E-05 | 2.45E-03 |
| adenyl ribonucleotide binding (GO:0032559) | 1467 | 90 | 1.89 | 1.67E-08 | 1.34E-05 |
| adenyl nucleotide binding (GO:0030554) | 1480 | 90 | 1.88 | 2.88E-08 | 1.73E-05 |
| kinase binding (GO:0019900) | 857 | 50 | 1.8 | 1.27E-04 | 1.90E-02 |
| cytoskeletal protein binding (GO:0008092) | 1010 | 57 | 1.74 | 9.49E-05 | 1.47E-02 |
| anion binding (GO:0043168) | 2312 | 129 | 1.72 | 2.57E-09 | 4.10E-06 |
| purine ribonucleoside triphosphate binding (GO:0035639) | 1723 | 96 | 1.72 | 5.03E-07 | 1.86E-04 |
| transition metal ion binding (GO:0046914) | 991 | 55 | 1.71 | 1.78E-04 | 2.59E-02 |
| carbohydrate derivative binding (GO:0097367) | 2174 | 120 | 1.7 | 2.03E-08 | 1.39E-05 |
| nucleoside phosphate binding (GO:1901265) | 2052 | 111 | 1.67 | 1.77E-07 | 8.49E-05 |
| nucleotide binding (GO:0000166) | 2052 | 111 | 1.67 | 1.77E-07 | 7.72E-05 |
| purine ribonucleotide binding (GO:0032555) | 1803 | 97 | 1.66 | 1.75E-06 | 4.42E-04 |
| purine nucleotide binding (GO:0017076) | 1817 | 97 | 1.65 | 2.66E-06 | 6.07E-04 |
| ribonucleotide binding (GO:0032553) | 1821 | 97 | 1.65 | 2.73E-06 | 5.96E-04 |
| small molecule binding (GO:0036094) | 2398 | 122 | 1.57 | 1.06E-06 | 3.40E-04 |
| enzyme binding (GO:0019899) | 2292 | 115 | 1.55 | 4.20E-06 | 8.75E-04 |
| ion binding (GO:0043167) | 5283 | 258 | 1.51 | 9.12E-13 | 4.37E-09 |
| cation binding (GO:0043169) | 3667 | 173 | 1.46 | 3.39E-07 | 1.35E-04 |
| metal ion binding (GO:0046872) | 3571 | 168 | 1.45 | 7.39E-07 | 2.53E-04 |
| catalytic activity, acting on a protein (GO:0140096) | 2254 | 105 | 1.44 | 2.21E-04 | 2.87E-02 |
| hydrolase activity (GO:0016787) | 2334 | 108 | 1.43 | 2.18E-04 | 2.91E-02 |
| catalytic activity (GO:0003824) | 5539 | 247 | 1.38 | 3.24E-08 | 1.73E-05 |
| protein binding (GO:0005515) | 9502 | 385 | 1.25 | 1.18E-08 | 1.13E-05 |
| binding (GO:0005488) | 13944 | 539 | 1.19 | 5.60E-12 | 1.34E-08 |
| transmembrane signaling receptor activity (GO:0004888) | 2162 | 38 | 0.54 | 2.60E-05 | 4.62E-03 |
| DNA-binding transcription factor activity (GO:0003700) | 1341 | 21 | 0.48 | 2.07E-04 | 2.84E-02 |
| DNA-binding transcription factor activity, RNA polymerase II-specific (GO:0000981) | 1282 | 20 | 0.48 | 2.88E-04 | 3.64E-02 |
| G protein-coupled receptor activity (GO:0004930) | 755 | 8 | 0.33 | 1.93E-04 | 2.72E-02 |
| olfactory receptor activity (GO:0004984) | 1134 | 11 | 0.3 | 1.24E-06 | 3.72E-04 |

Appendix Table S5. PANTHER version 16 GO cellular component overrepresentation results for the 709 genes identified by outlier analysis (Appendix Table S1).

| Analysis Type: | PANTHER Overrepresentation Test (Released 20210224) | | | | |
| --- | --- | --- | --- | --- | --- |
| Annotation Version and Release Date: | GO Ontology database DOI: 10.5281/zenodo.5228828 Released 2021-08-18 | | | | |
| Analyzed List: | Client Text Box Input (Mus musculus) | | | | |
| Reference List: | Mus musculus (all genes in database) | | | | |
| Test Type: | FISHER | | | | |
| Correction: | FDR | | | | |
| GO cellular component complete | Mus musculus - REFLIST (21988) | Client Text Box Input (712) | Client Text Box Input (fold Enrichment) | Client Text Box Input (raw P-value) | Client Text Box Input (FDR) |
| spindle microtubule (GO:0005876) | 68 | 10 | 4.54 | 1.59E-04 | 9.47E-03 |
| dynein complex (GO:0030286) | 57 | 8 | 4.33 | 9.39E-04 | 4.10E-02 |
| synaptic vesicle (GO:0008021) | 215 | 19 | 2.73 | 1.58E-04 | 9.72E-03 |
| exocytic vesicle (GO:0070382) | 238 | 19 | 2.47 | 7.81E-04 | 3.65E-02 |
| transport vesicle (GO:0030133) | 325 | 24 | 2.28 | 4.68E-04 | 2.56E-02 |
| microtubule (GO:0005874) | 426 | 31 | 2.25 | 6.08E-05 | 5.69E-03 |
| spindle (GO:0005819) | 330 | 24 | 2.25 | 5.14E-04 | 2.59E-02 |
| collagen-containing extracellular matrix (GO:0062023) | 393 | 28 | 2.2 | 1.90E-04 | 1.10E-02 |
| lytic vacuole (GO:0000323) | 524 | 37 | 2.18 | 2.22E-05 | 2.91E-03 |
| lysosome (GO:0005764) | 524 | 37 | 2.18 | 2.22E-05 | 2.73E-03 |
| **extracellular matrix (GO:0031012)** | 523 | 35 | 2.07 | 1.08E-04 | 8.18E-03 |
| external encapsulating structure (GO:0030312) | 525 | 35 | 2.06 | 1.14E-04 | 8.27E-03 |
| neuron to neuron synapse (GO:0098984) | 391 | 26 | 2.05 | 9.21E-04 | 4.11E-02 |
| vacuole (GO:0005773) | 618 | 40 | 2 | 8.74E-05 | 7.80E-03 |
| microtubule cytoskeleton (GO:0015630) | 1263 | 80 | 1.96 | 4.10E-08 | 1.01E-05 |
| dendrite (GO:0030425) | 738 | 43 | 1.8 | 3.48E-04 | 1.95E-02 |
| dendritic tree (GO:0097447) | 741 | 43 | 1.79 | 5.03E-04 | 2.67E-02 |
| polymeric cytoskeletal fiber (GO:0099513) | 683 | 39 | 1.76 | 1.02E-03 | 4.37E-02 |
| somatodendritic compartment (GO:0036477) | 1053 | 59 | 1.73 | 8.96E-05 | 7.65E-03 |
| cytoskeleton (GO:0005856) | 2247 | 125 | 1.72 | 5.23E-09 | 1.71E-06 |
| supramolecular polymer (GO:0099081) | 917 | 50 | 1.68 | 6.18E-04 | 3.04E-02 |
| plasma membrane region (GO:0098590) | 1253 | 68 | 1.68 | 5.96E-05 | 5.85E-03 |
| supramolecular fiber (GO:0099512) | 910 | 49 | 1.66 | 8.47E-04 | 3.87E-02 |
| cell projection (GO:0042995) | 2597 | 139 | 1.65 | 7.54E-09 | 2.12E-06 |
| synapse (GO:0045202) | 1346 | 72 | 1.65 | 5.37E-05 | 5.56E-03 |
| supramolecular complex (GO:0099080) | 1236 | 66 | 1.65 | 1.47E-04 | 9.65E-03 |
| plasma membrane bounded cell projection (GO:0120025) | 2357 | 120 | 1.57 | 1.21E-06 | 1.99E-04 |
| neuron projection (GO:0043005) | 1538 | 78 | 1.57 | 1.46E-04 | 9.91E-03 |
| cell junction (GO:0030054) | 1990 | 100 | 1.55 | 2.24E-05 | 2.59E-03 |
| cytosol (GO:0005829) | 3804 | 190 | 1.54 | 8.32E-10 | 4.09E-07 |
| vesicle (GO:0031982) | 2178 | 104 | 1.47 | 1.04E-04 | 8.21E-03 |
| intracellular vesicle (GO:0097708) | 2036 | 95 | 1.44 | 5.13E-04 | 2.65E-02 |
| cytoplasmic vesicle (GO:0031410) | 2032 | 94 | 1.43 | 6.51E-04 | 3.12E-02 |
| intracellular non-membrane-bounded organelle (GO:0043232) | 4381 | 197 | 1.39 | 1.18E-06 | 2.32E-04 |
| non-membrane-bounded organelle (GO:0043228) | 4381 | 197 | 1.39 | 1.18E-06 | 2.11E-04 |
| endomembrane system (GO:0012505) | 4079 | 175 | 1.32 | 9.10E-05 | 7.45E-03 |
| cytoplasm (GO:0005737) | 11382 | 480 | 1.3 | 1.08E-16 | 2.12E-13 |
| cell periphery (GO:0071944) | 5995 | 247 | 1.27 | 1.93E-05 | 2.71E-03 |
| plasma membrane (GO:0005886) | 5494 | 224 | 1.26 | 1.32E-04 | 9.27E-03 |
| protein-containing complex (GO:0032991) | 5486 | 223 | 1.26 | 1.58E-04 | 1.00E-02 |
| intracellular organelle (GO:0043229) | 12492 | 487 | 1.2 | 4.98E-10 | 3.26E-07 |
| organelle (GO:0043226) | 12793 | 495 | 1.19 | 8.83E-10 | 3.47E-07 |
| intracellular anatomical structure (GO:0005622) | 14145 | 547 | 1.19 | 1.74E-12 | 1.71E-09 |
| membrane-bounded organelle (GO:0043227) | 11894 | 446 | 1.16 | 6.22E-06 | 9.40E-04 |
| intracellular membrane-bounded organelle (GO:0043231) | 11439 | 427 | 1.15 | 3.15E-05 | 3.44E-03 |
| cellular anatomical entity (GO:0110165) | 19025 | 659 | 1.07 | 7.09E-07 | 1.55E-04 |
